# The effects of supplementation of vitamin D to the egg‐yolk extender on cryopreservation of ram semen

**DOI:** 10.1002/vms3.1526

**Published:** 2024-07-04

**Authors:** Ahmet Aktar, Mehmed Berk Toker, Davut Koca, Umit Can Uzun, Selim Alcay

**Affiliations:** ^1^ Faculty of Veterinary Medicine, Department of Reproduction and Artificial Insemination Bursa Uludag University Bursa Turkey; ^2^ Faculty of Veterinary Medicine, Department of Obstetrics and Gynecology Yuzuncu Yil University Van Turkey

**Keywords:** cryopreservation, flow cytometer, ram, semen, vitamin D

## Abstract

**Objectives:**

This study aimed to examine the effects of supplementation of vitamin D to the egg‐yolk extender on characteristics of frozen–thawed ram semen.

**Methods:**

Semen samples obtained from adult rams were pooled and divided into five equal volumes. It was reconstituted with extenders containing different concentrations of vitamin D: 0 (control), 12.5 (VITD 12.5), 25 (VITD 25), 50 (VITD 50), and 100 ng/mL (VITD 100), and then they were frozen. Sperm motility parameters, plasma membrane functional integrity, acrosomal integrity, DNA fragmentation, and mitochondrial membrane potential of the groups were evaluated after sperm thawing.

**Results:**

Total motility and progressive motility were higher in VITD 50 than in all other groups (*p* < 0.05). Higher sperm straightness, linearity, and wooble were higher in VITD 50 than in the control group (*p* < 0.05). A similar pattern of VITD 50 was observed for plasma membrane integrity and mitochondrial membrane potential (*p* > 0.05).

**Conclusions:**

In the study, it was observed that adding vitamin D to the extender had a beneficial effect on ram spermatological parameters. In addition, it was concluded that the use of the 50 ng/mL vitamin D in the extender provided more effective protection than the other doses.

## Graphical abstract

This study aimed to examine the effects of supplementation of vitamin D to the egg‐yolk extender on characteristics of frozen–thawed ram semen. Sperm motility, plasma membrane functional integrity, acrosomal integrity, DNA fragmentation, and mitochondrial membrane potential of the groups were determined. In the study, it was observed that adding vitamin D to the extender had a beneficial effect on ram spermatological parameters. In addition, it was concluded that the use of the 50 ng/mL vitamin D in the extender provided more effective protection than the other doses.

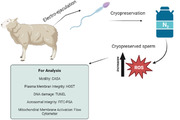

## 1 INTRODUCTION

Nowadays, the success achieved in artificial insemination applications with frozen ram semen is not satisfactory. One of the most important reasons for this is intracellular ice crystallization, cold shock, oxidative stress, and reactive oxygen species (ROS) that occur as a result of freezing–thawing, as in other species. Depending on these, sperm motility, mitochondrial injury, DNA fragmentation, viability and fertilization capacity of spermatozoa decrease (Agarwal et al., 2014; Desai et al., 2009; Moghadam et al., 2020). To eliminate all these problems and further the success achieved, it is possible to freeze the semen for a long time by using different chemicals and antioxidants (Trounson, 1990).

Vitamin D, which is a prohormone in steroid structure, exists in two forms, D3 and D2 (Gil et al., 2018). A large part of the D3 form is synthesized in the skin by ultraviolet‐B rays, whereas a very small part is taken from the diet (Gil et al., 2018; Uitterlinden et al., 2004). The D2 form is provided from plant‐based foods. There are results from experimental animal studies that show vitamin D regulates testicular function. Moreover, the vitamin D receptor (VDR) and its metabolizing enzymes are expressed in germ cells and mature spermatozoa (Jensen et al., 2010).

In addition to its effects on the immune system, it is known to have a protective effect against oxidative stress (Berridge, 2015; Dong et al., 2012). This effect is indicated by binding to the VDR, which is a nuclear receptor (Berridge, 2015). Oxidative stress occurs as a result of the increase in free radicals, especially ROS, and the deterioration of the balance between antioxidants that detoxify prooxidants (Grygiel‐Gorniak & Puszczewicz, 2014). The reactions that form ROS under physiological conditions are reversible, and after an oxide protein completes its function, it is reduced to its inactive form. Vitamin D regulates the expression of antioxidant genes that prevent oxidative stress through transcription factors such as Klotho and nuclear factor erythroid 2–related factor 2, by removing ROS or reversing the oxidative changes that occur in the normal ROS signalling process (Berridge, 2015). Vitamin D increases the expression of gamma‐glutamyl transpeptidase, which increases glutathione (GSH) synthesis, and increased GSH synthesis decreases NOX (NADPH oxidase) expression, which is one of the main sources of ROS production (Dong et al., 2012). Vitamin D also increases the production of GSH, which is the major redox buffer, by increasing the expression of antioxidant enzymes, such as glucose 6 phosphate hydrogenase, glutamate cysteine ligase and GSH reductase; by increasing the expression of GSH peroxidase, it provides the conversion of H_2_O_2_ to water (Briones and Darwish, 2014; Jain and Micinski, 2013).

Although some studies have shown that vitamin D has significant effects on the cryopreservation of human, bull, and pig semen, to our best knowledge, no study has been conducted on ram semen. In our study, it was aimed to examine the effects of different ratios of vitamin D supplementation to the extender on the spermatological parameters of the frozen/thawed ram semen.

## 2 MATERIALS AND METHODS

The study was carried out with the scientific ethics committee document approved by Bursa Uludag University Committee of Ethics (Approval no: 2021‐13/03). Also, the work was completed with chemicals from Merck and Sigma.

### 2.1 Experimental design

This planned study was conducted to investigate the effects of different doses of vitamin D (Sigma (St. Louis, MO, USA)) added to the extender used for the frozen storage of ram semen. Taking into account the previous works, vitamin D doses were determined in groups as no vitamin D (control), 12.5 (VITD 12.5), 25 (VITD 25), 50 (VITD 50) and 100 ng/mL (VITD 100). Each trial was repeated five times under the same conditions.

### 2.2 Semen collection, extender preparation, and dilution

Six different Merinos rams under the same care and feeding conditions were used to collect fresh semen. Semen samples were collected twice a week for 3 weeks using an electro‐ejaculation method (Ruakura Goat Probe Plastic Products, Hamilton, New Zealand), and then ejaculates were kept in a water bath at 37°C at the laboratory. After an initial evaluation, ram semen samples having motility >80%, viability 85%, rapid wave >+3, and ≥1.5 × 10^9^ spermatozoa/mL were used to categorize semen.

Semen extenders 223.7 mmol/L Tris, 66.6 mmol/L citric acid, 55.5 mmol/L fructose, 4.03 mmol/L EDTA, 4 g/L penicillin G and 100.4 mmol/L trehalose contain 3 g/L dihydrostreptomycin and 20% egg yolk. According to the experimental design, each group was supplemented with vitamin D. Vitamin D3 (Vitamin D) was dissolved in ethanol to achieve concentrations of 12.5, 25, 50, and 100 ng/mL for the experimental groups. Then, distilled water was used to dilute it to obtain the desired doses.

All in all, ejaculates from rams were pooled and split into five equal aliquots. They were diluted to a final concentration of 200 × 10^6^ (spermatozoon/mL) with extenders having no vitamin D (control), 12.5 (VITD 12.5), 25 (VITD 25), 50 (VITD 50) and 100 ng/mL (VITD 100). They were then gradually cooled to 4°C and equilibrated at 4°C for 2 h.

### 2.3 Semen freezing and thawing

As in a previous study that was conducted by Alcay et al. (2022), equilibrated semen was placed into 0.25 mL straws, and they were cooled in liquid nitrogen vapour at 3°C/min from 5 to −8°C and at 25°C/min cooling rate using Nicool Plus PC (Air Liquide, Marne‐la‐Vallée Cedex 3). After that, they were cooled from −8 to −120°C. The straws were then plunged into liquid nitrogen at −196°C, where they were stored for at least 1 month. At least three straws from each group were thawed at 37°C for 30 s in a water bath to evaluate post‐thaw semen characteristics.

### 2.4 Examination of frozen sperm

After thawing, sperm motility and kinematic parameters (a computer‐assisted sperm analyser [CASA]), plasma membrane integrity (hypoosmotic swelling test [HOST]), DNA damage (TUNEL), acrosomal integrity (FITC‐PSA) and mitochondrial membrane activation potential (CytoFLEX Flow Cytometer (5,5′,6,6′‐tetrachlor1,1′3,3′‐tetramethyl benzimidazolylcarbocyanine iodide [(JC‐1)‐PI]) were evaluated. All of the applications were made by the same person from start to finish.

#### 2.4.1 Sperm motility and kinematic parameters

Ram sperm motility and kinematic parameters consideration were executed using a CASA (Ceros II, IMV Technologies) using leja slides that were pre‐heated to 37°C. Total motility (%), rapid motility (%), progressive motility (%), velocity average path (VAP, µm/s), velocity straight line (VSL, µm/s), velocity curve linear (VCL, µm/s), straightness (STR, VSL/VAP × 100), linearity (LIN, VSL/VCL × 100) and wobble (WOB, %) were assessed. For each sample, 250–400 sperms were evaluated in 8 fields (Inanc et al., 2019).

#### 2.4.2 Plasma membrane integrity

Plasma membrane integrity was tested using the HOST. This method was conducted as Alcay et al. (2015) indicated before, and sperms with a curled tail were counted and evaluated.

#### 2.4.3 Fragmentation of DNA

For evaluation, 10 µL of sperm samples were placed in 100 µL of PBS and then centrifuged at 2500 rpm for 5 min. The supernatant was discarded, and 75 µL of PBS was added and centrifuged again. Then, a drop was smeared on a slide and fixed with 10% formaldehyde for 20 min. After fixation, the slides were washed with PBS and then dried. Prepared slides were incubated for 1 h at 37°C with a TUNEL reaction mixture containing terminal deoxynucleotidyl transferase plus dUTP tag. TUNEL‐positive sperm cells were first detected under a fluorescent microscope, and then the total number of cells in the area was determined under a phase contrast microscope.

#### 2.4.4 Acrosomal integrity

Acrosomal integrities of sperm cells were evaluated by the FITC‐conjugated Pisum sativum agglutinin (FITC‐PSA) method (Alcay et al., 2022); sperm sample of 10 µL was added to 100 µL of PBS and centrifuged. The supernatant was discarded, and then 100 µL of PBS was added and centrifuged again. After the centrifugation, a drop from the pellet was smeared on the slide. Then the prepared slides were fixed with acetone. After staining, 200 spermatozoa cells were evaluated under a fluorescent attachment microscope (Alcay et al., 2015).

#### 2.4.5 Mitochondrial membrane activation potential

Mitochondrial membrane potential assessment was performed with a flow cytometer analyser, CytoFlex Flow Cytometer (Beckman Coulter). Sperm samples were evaluated with a 488 nm blue laser beam (50 mW laser output), emission filters of 525 ± 40, 585 ± 42, and 610 ± 20, and data were taken from 10,000 events.

**FIGURE 1:**
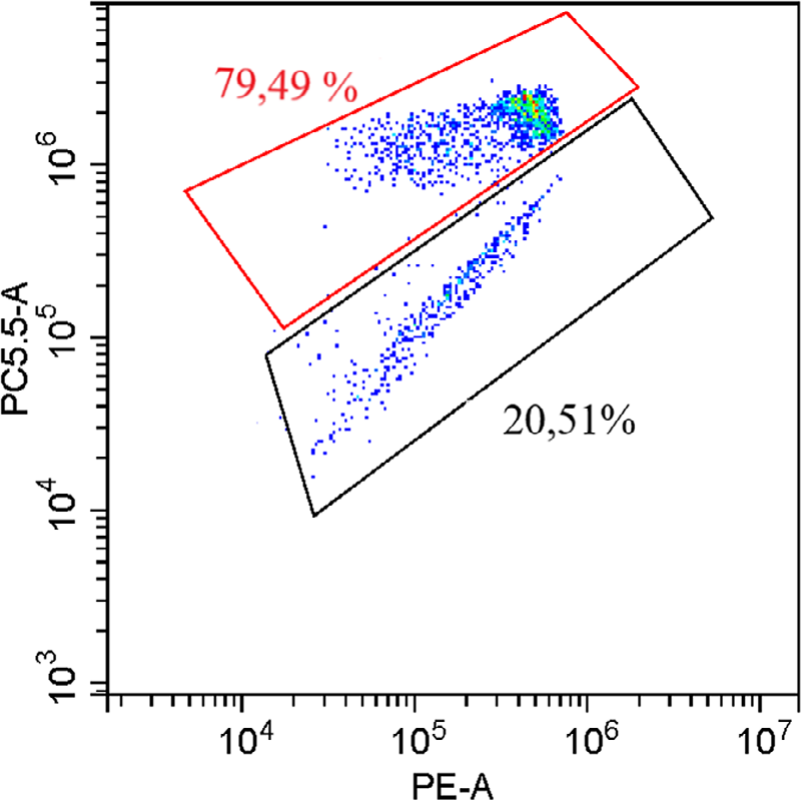
Flow cytometric examination of frozen–thawed ram sperm stained with JC‐1. Red area = high mitochondrial membrane potential; JC‐1 = 5,5′,6,6′‐tetrachloro1,1′3,3′‐tetramethylbenzimidazolylcarbocyanine iodide; black area = low mitochondrial membrane potential; PE‐A = propidium iodide axis; PC5.5‐A JC‐1 axis.

Frozen–thawed spermatozoa were determined with 5,5′,6,6′‐tetrachloro1,1′3,3′ tetramethyl benzimidazolylcarbocyanine iodide (JC‐1)‐PI molecular probes. Straws were thawed at 37°C; JC‐1 of 10 µL and 3 µL of PI were added to 10 µL of sperm sample in 487 µL Tris solution. It was then incubated at 37°C for 30 min (in a dark environment). Damaged and intact spermatozoa were determined by PI. Following this, cells with high mitochondrial membrane potential with JC‐1 fluorescent orange and cells with low mitochondrial membrane potential are fluorescent green (Inanc et al., 2019) (Figure 1).

### 2.5 Statistical analysis

SPSS 28 was used for statistical analysis in the study. First, the normality test Shapiro–Wilk was used, and the distributions were determined. The ANOVA test and Kruskal–Wallis test were applied according to the normality of the distribution. The groups with differences were determined by the Tukey and Mann–Whitney *U* tests, respectively. All obtained data were given as mean ± standard deviation, and *p*‐values less than 0.05 were considered to be significantly different.

## 3 RESULTS

Twelve sperm parameters were considered in the post‐frozen–thawed evaluation in this study. The total motility and progressive motility were higher (*p* < 0.05) in VITD 50 than in all other groups. STR, LIN, and WOB were higher (*p* < 0.05) in VITD 50 than in the control group. Inversely, lower values (*p* < 0.05) of STR, LIN, and WOB were observed in VITD 100 than in the control group and some other treated groups (Table 1). A higher (*p* < 0.05) mitochondrial membrane potential was observed in VITD 50 than in all other groups (Table 2). Plasma membrane integrity is higher (*p* < 0.05) in VITD 25 and VITD 50 than in all other groups.

**TABLE 1:** The mean of studied ram sperm post‐thawing motility and kinematic parameters on different extender groups.

Kinematics/Groups	Control	VITD 12.5 (ng/mL)	VITD 25 (ng/mL)	VITD 50 (ng/mL)	VITD 100 (ng/mL)
**VAP (µm/s)**	84.87 ± 23.33^a^	89.70 ± 16.16^a^	101.36 ± 21.14^a^	109.39 ± 21.82^a^	91.68 ± 18.15^a^
**VSL (µm/s)**	65.96 ± 18.07^a^	68.27 ± 10.32^a^	76.38 ± 14.19^a^	79.01 ± 14.95^a^	61.46 ± 13.70^a^
**VCL (µm/s)**	159.21 ± 46.27^a^	173.12 ± 41.72^a^	192.76 ± 50.70^a^	227.34 ± 45.85^a^	198.40 ± 31.24^a^
**STR (%)**	70.38 ± 2.58^a^	76.87 ± 6.60^a,b^	76.65 ± 4.85^a,b^	74.72 ± 1.65^b^	68.71 ± 1.77^c^
**LIN (%)**	41.17 ± 5.12^a^	45.80 ± 11.44^a,b^	45.81 ± 9.04^a,b^	47.83 ± 2.13^b^	35.27 ± 1.03^c^
**WOB (%)**	54.41 ± 4.34^a^	56.84 ± 9.29^a,c^	57.46 ± 8.10^a^	58.42 ± 1.36^b,c^	48.94 ± 0.51^b^
**Total motility (%)**	37.00 ± 6.70^a^	43.00 ± 5.70^a^	44.00 ± 4.18^a^	55.00 ± 2.00^b^	45.00 ± 3.53^a^
**Progressive motility (%)**	20.80 ± 3.56^a^	26.00 ± 4.32^a^	29.20 ± 3.41^a^	40.20 ± 4.51^b^	27.50 ± 3.25^a^

*Note*: Data are presented in mean ± S.D. Different letters within the same rows show significant differences among the groups (*p* < 0.05).

Abbreviations: LIN, linearity VSL/VCL × 100; STR, straightness VSL/VAP × 100; VAP, velocity average path; VCL, velocity curve linear; VITD, vitamin D; VSL, velocity straight line; WOB, wobble VAP/VCL × 100.

**TABLE 2:** The mean of studied ram sperm post‐thawing parameters on different extender groups.

Groups	Control	VITD 12.5 (ng/mL)	VITD 25 (ng/mL)	VITD 50 (ng/mL)	VITD 100 (ng/mL)
**DNA fragmentation (%)**	13.60 ± 5.72^a^	12.80 ± 5.58^a^	14.40 ± 9.12^a^	16.00 ± 4.72^a^	15.80 ± 5.01^a^
**Acrosomal integrity (%)**	70.00 ± 4.89^a^	70.00 ± 2.34^a^	75.00 ± 4.24^a^	74.00 ± 2.64^a^	74.40 ± 3.36^a^
**Host (%)**	83.60 ± 3.78^a^	82.40 ± 1.81^a^	88.00 ± 0.70^b^	87.40 ± 2.19^b^	84.80 ± 2.38^a^
**HMMP (%)**	72.88 ± 15.73^a^	69.41 ± 24.11^a^	68.39 ± 20.39^a^	79.49 ± 6.48^b^	64.12 ± 25.15^a^

*Note*: Data are presented in mean ± S.D. Different letters within the same rows show significant differences among the groups (*p* < 0.05).

Abbreviations: HMMP, high mitochondrial membrane potential; Host, plazma membrane integrity; VITD, vitamin D.

## 4 DISCUSSION

The aim of this study was to investigate whether vitamin D protects sperm cells against harmful effects exposed during the cryopreservation process. Due to this feature, vitamin D was added to diluents in different doses as the main materials, and their effects were investigated.

Cryopreservation of sperm ensures the long‐term preservation of genetic material. However, it is known that freezing has negative effects on the fertilization ability of semen (Teymoori et al., 2024). These undesirable effects are shown by reducing sperm viability, motility, plasma membrane integrity, acrosomal integrity, DNA fragmentation, and mitochondrial membrane potential (Donnelly et al., 2000).

Motility and progressiveness are one of the basic quality parameters in the evaluation of semen. In the present study, it was observed that the addition of vitamin D increased the motility rate of ram semen after frozen thawing. The total motility rate of VITD 50, which is one of the vitamin D groups added to the extenders at different rates, is significantly higher compared to the other groups (*p* < 0.05). When the kinematic parameters are examined, there is no significant difference among the groups in VAP, VSL, and VCL values. In STR, LIN and WOB values, VITD 12.5, VITD 25 and VITD 50 added groups were significantly different from the VITD 100 group. Considering these results, the results of this study show parallelism with other Holstein and human studies. (Asadpour et al., 2021; Moghadam et al., 2020; Moghadam et al., 2019).

Spermatozoon plasma membrane integrity is positively related to the capacitation of sperm cells, which has a very important effect on acrosome reaction and sperm fusion with the egg during fertilization (Ustuner et al., 2016). However, the integrity of the spermatozoon plasma membrane is adversely affected by the cryopreservation process (Emamverdi et al., 2013; Forouzanfar et al., 2010; Najafi et al., 2013). The known negative effects are the effects of cold shock, ice crystallization, osmotic stress and lipid peroxidation formed during cryopreservation on sperm membrane permeability and morphology (El‐Kon, 2011; Taylor et al., 2009). Therefore, it is very important to maintain membrane integrity during the freezing process to prevent cellular damage. HOST is an optimized test to detect subtle changes in spermatozoon membrane functionality (Maxwell & Salamon, 1993). In our study, it was observed that vitamin D preserved the integrity of the plasma membrane after thawing. In the present study, VITD 25 and VITD 50 are statistically higher than the other groups. (*p* < 0.05) These data are in agreement with other human and ram mentioned studies.

Acrosomal defect and DNA fragmentation are one of the undesirable effects of the cryopreservation process (Nur et al., 2010). These effects are caused by ROS emerging after cryopreservation. Therefore, the extenders used for cryopreservation should protect the semen against freezing (Kumar et al., 2015). The addition of vitamin D has a positive effect on the ROS, and results of some human studies support this claim (Jain & Micinski, 2013; Moghadam et al., 2019). Vitamin D indicates these effects by binding to nuclear receptors against oxidative stress (Chen & Zhi, 2020; Wang et al., 2017). They express antioxidant genes that prevent oxidative stress by eliminating ROS or reversing the normal ROS signalling process (Mokhtari et al., 2017). In our study, vitamin D has positive effects against acrosomal damage and DNA fragmentation. In our study, the lowest acrosomal damage and DNA fragmentation levels were obtained in the VITD 25 group.

There is a positive correlation between mitochondrial membrane potential and plasma membrane integrity and motility. Mitochondria have axonemes that produce intracellular ATP (Gürler et al., 2016; Inanc et al., 2019). When these are damaged as a result of cryopreservation, ATP production and motility are reduced. Sperm plasma membrane functional integrity is an important factor for oocytes fusion of sperm. Therefore, the evaluation of plasma membrane integrity is crucial for sperm quality (Maxwell & Salamon, 1993). In addition, plasma membrane permeability and functional integrity are important for the capacitation and acrosome reactions of the spermatozoon. During the freeze–thaw process, cold shock and lipid peroxidation damage the sperm membrane permeability and integrity. Increasing the fluidity of plasma membrane is essential for protection against cold (Fang et al., 2014). In our study, the mitochondrial membrane potential of the VITD 50 group was higher than the other groups and the control group, and there was a significant difference (*p* < 0.05). However, in plasma membrane integrity, VITD 25 and VITD 50 were found to be significantly different from the control group and other groups (*p < 0.05*). The HOST and mitochondrial membrane potential values of our study are in good agreement with previous goat studies (Alcay et al., 2016; Salmani et al., 2014).

As a result of all parameters checked after cryopreservation, it was observed that the addition of vitamin D to the extender can protect the sperm against the harmful effects of cryopreservation. The semen protection effect was consistently higher in VITD 50 than in the control group and when compared with other groups. In future studies, post‐incubation parameters and the effect of vitamin D on different animal species should be examined. In addition, semen frozen with vitamin D should be used for reproduction, and its success should be evaluated.

## AUTHOR CONTRIBUTIONS

**Ahmet Aktar, M. Berk Toker and Davut Koca**: Development of methodology; preparation of the draft (first version); collection; analysis and interpretation of data. **Selim Alcay**: Formal analysis; supervision; validation; funding acquisition; project administration; visualization of the writing of the manuscript. **Davut Koca**: Conceptualization; supervision; investigation; visualization; writing—review and editing. **Umit Can Uzun**: writing—original draft; writing—review and editing; visualization. **Ahmet Aktar**: Validation; conceptualization; investigation; visualization; writing—review and editing; approval of the final version to be published.

## CONFLICT OF INTEREST STATEMENT

The authors declare no conflicts of interest.

## ETHICS STATEMENT

All procedures used were approved by the Bursa Uludag University Committee of Ethics (Approval No.: 2021‐13/03).

## Data Availability

The data that support the findings of this study are available from the corresponding author upon reasonable request.
